# Bioinspired BG/PPDO-CNP/Pluronic F-127 hydrogel fibrous dura mater with temporally programmed degradation and adhesion prevention inspired by the hierarchical architecture of dragonfly wings

**DOI:** 10.1093/rb/rbag154

**Published:** 2026-07-22

**Authors:** Junqin Mao, Heng Zheng, Qiuyu Zeng, Wanli Zhang, Qiang Liu, Tao Shen, Xinyu Zhang, You Tang, Wei Tang, Guoyu Lv

**Affiliations:** College of Physics, Sichuan University, Chengdu 610065, China; College of Physics, Sichuan University, Chengdu 610065, China; College of Physics, Sichuan University, Chengdu 610065, China; College of Physics, Sichuan University, Chengdu 610065, China; College of Physics, Sichuan University, Chengdu 610065, China; College of Physics, Sichuan University, Chengdu 610065, China; College of Physics, Sichuan University, Chengdu 610065, China; College of Physics, Sichuan University, Chengdu 610065, China; College of Physics, Sichuan University, Chengdu 610065, China; College of Physics, Sichuan University, Chengdu 610065, China

**Keywords:** dural repair, time-programmed, Co-ESP, poly(p-dioxanone)

## Abstract

Inspired by the hierarchical structure of dragonfly wings, a bioinspired double-layer artificial dura mater (CBPP) integrating poly(p-dioxanone) (PPDO), bioactive glass (BG), and cerium oxide nanoparticles (CNP) within a Pluronic F-127 hydrogel was developed to achieve temporally programmed degradation and dynamic bioactivity. The outer PPDO layer provided mechanical strength, while the inner BG/CNP-loaded hydrogel fibrous layer offered reduction of adhesion, as well as antioxidant and angiogenic functions. *In vitro* studies demonstrated that the CBPP-2 group exhibited excellent cytocompatibility, Reactive Oxygen Species (ROS)-scavenging capacity, and sequential immunomodulation, leading to accelerated tissue repair. *In vivo* implantation confirmed that CBPP-2 promoted neodura formation, enhanced angiogenesis, and minimized fibrosis and tissue adhesion. The degradation timeline of CBPP closely matched the physiological repair process, coupling early anti-inflammatory activity with later tissue regeneration. This bioinspired, time-responsive scaffold provides a promising strategy for dural substitutes and offers valuable insights into temporal tissue regeneration.

## Graphical Abstract

**Graphical Abstract:**
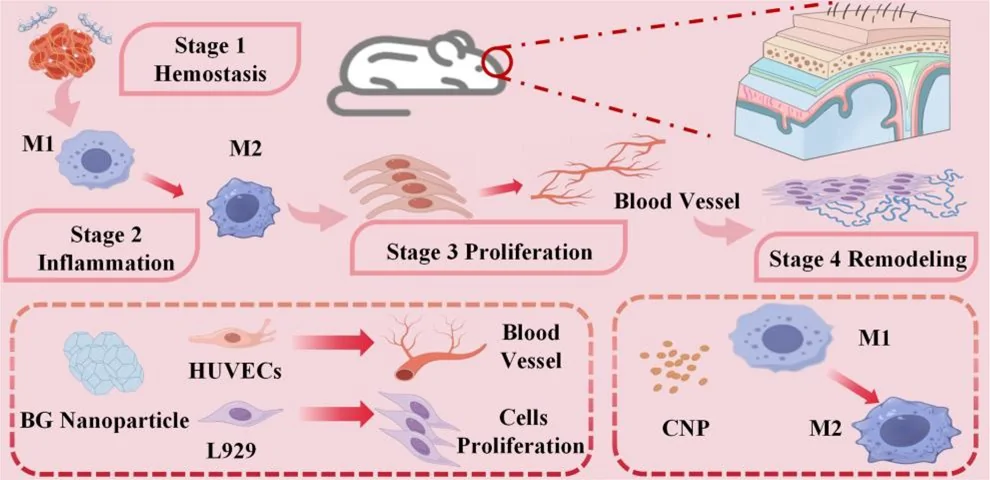

## Introduction

Dura mater, a double-layer membrane enveloping the central nervous system, plays a crucial role in maintaining intracranial homeostasis and protecting neural tissue [1]. During neurosurgical procedures, the dura mater is highly susceptible to defects caused by trauma, tumor invasion, or surgical manipulation. Effective repair of dural defects remains a major challenge in neurosurgery [2], as inadequate reconstruction is strongly linked to postoperative central nervous system complications [3–6]. Therefore, developing dura substitutes that can promote tissue repair, reduce postoperative cerebrospinal fluid leakage, and prevent adhesion formation is highly important for enhancing surgical safety and improving patient outcomes [7–12].

Dural repair proceeds through temporally defined stages: defect formation, inflammation, angiogenesis, and remodeling. Early inflammation (Days 1–7) involves ROS accumulation, where excessive responses hinder healing [13–15]. The proliferative phase (Days 7–14) features angiogenesis and microvascular formation, followed by fibroblast-driven collagen remodeling after Day 14 to restore dural integrity [16, 17]. Various dural substitutes have been explored, including autografts, allografts, natural biomaterials, and synthetic polymers. Although synthetic polymers offer better controllability and availability, current materials such as PLLA (poly-L-lactic acid), PCL (polycaprolactone), and PU (polyurethane) degrade too slowly [18–20], often causing chronic inflammation and insufficient mechanical stability. Their limited bioactivity also hinders integration and regeneration, while the mismatch between degradation timing and tissue repair highlights the need for next-generation substitutes with coordinated biofunctionality [21–25].

An ideal artificial dura should perform distinct yet coordinated roles across these phases. Emerging biomaterial strategies aim to integrate immunomodulatory functions for dynamic regeneration. Cerium oxide nanoparticles (CNP) act as redox-active nanozymes, cyclically scavenging ROS via Ce^3+^/Ce^4+^ transitions, reducing oxidative stress, and promoting macrophage polarization from M1 to M2 phenotypes, thus shortening inflammation and promoting repair [26, 27]. Du *et al.* successfully utilized CNP nanoparticles to promote macrophage polarization in diabetic wound applications [28, 29], demonstrating that CNP nanoparticles serve as an effective approach for shortening the early inflammatory stage [29]. Meanwhile, bioactive glass (BG) releases Si^4+^ and Ca^2+^ ions that enhance endothelial proliferation and vascularization, accelerating defect closure [30–33]. Therefore, BG can effectively accelerate dura repair in the proliferative phase following the inflammatory response by stimulating angiogenesis and delivering essential substances for tissue repair, such as oxygen and nutrients. During the collagen remodeling phase, the artificial scaffold fabricated via electrospinning provides cell attachment sites, effectively preventing localized abnormal proliferation caused by the limitations of self-healing [34–38]. Electrospun poly(p-dioxanone) (PPDO) fibers offer high porosity, tunable degradation rates, and excellent mechanical flexibility, closely mimicking the extracellular matrix (ECM) [39, 40]. Their nanoscale architecture supports efficient cell adhesion, migration and nutrient exchange, making PPDO an ideal candidate for dura mater scaffolds requiring both structural integrity and biofunctionality [30, 41]. Mao *et al.* [41] demonstrated in a rat dural defect model that untreated defects typically resulted in the formation of disorganized fibrous tissue at the injury site. In contrast, the use of electrospun PPDO alone as a repair material enabled the formation of a relatively well-organized neo dura mater within 28 days. Meanwhile, F-127 is a hydrophilic and hydrophobic monomer that coexists to form block copolymers, capable of forming ordered structures in solution, with micelles being the most common. F-127 can self-assemble and gelatinize at the body temperature of 37°C. This property enables these systems to encapsulate and deliver small-molecule drugs, biomacromolecules, and other nanocarriers to their designed sites of action. Additionally, the contraction of the hydrophilic end is expected to significantly reduce tissue adhesions caused by dural defects [42–45]. Combining these mechanisms enables stage-specific modulation by suppressing inflammation early, stimulating angiogenesis mid-phase, and supporting matrix remodeling later.

Based on this concept, this work designed a double-layer artificial dura mater (CBPP) that unites adhesion prevention with time-programmed bioactivity [46]. The dense outer PPDO layer provides strength and sealing. The inner bioinspired fibrous layer mimics the dragonfly wing’s hierarchical structure [47], which is fabricated by electrospinning PPDO/BG and electrostatic spraying of CNP-loaded Pluronic F-127 hydrogel simultaneously. This configuration replicates the natural dura’s dense-porous architecture, offering both mechanical strength and a bioactive microenvironment. Sustained ion release from BG promotes vascular ingrowth, while CNP regulates ROS and inflammation, guiding a time-programmed transition from immune modulation to tissue regeneration.

The design strategy proposed in this study provides an emerging strategy for next-generation dura mater substitutes. This bioinspired, temporally responsive architecture not only mimics the multiscale hierarchical optimization of the dragonfly wing in structure but also integrates mechanical protection with dynamic cellular modulation in function. Just as the dragonfly wing adapts to aerodynamic fluctuations to maintain flight stability, the dura mater can dynamically respond to the evolving intracranial microenvironment, aligning its degradation and bioactivity with the tissue healing timeline. By guiding regeneration through a temporal gradient, this biomimetic scaffold demonstrates remarkable potential to reduce postoperative complications, such as adhesion to brain tissue, and enhance regenerative outcomes, offering an excellent solution for clinical neurosurgical dural reconstruction.

### Materials and methods

The artificial dura mater samples were fabricated according to the following procedure. 1.2 g PPDO was dissolved in 20 mL of hexafluoroisopropanol under sealed, light-protected conditions with stirring at room temperature for 4 h. The mixture was degassed under vacuum for 30 min to remove bubbles, yielding a 6 mg/mL PPDO solution. Portions (10 mL each) were poured into 10 cm glass Petri dishes at 30 min intervals, ensuring a uniform surface with a coating blade. The films were dried under vacuum at 37°C for 36 h to form uniform PPDO base membranes. Next, 2.4 g of PPDO and an appropriate amount of 45S5 BG extract were dissolved in 30 mL of hexafluoroisopropanol under continuous stirring for 4 h to obtain a PPDO spinning solution with a concentration of 0.08 g/mL. The silicon concentration in Solution 1 was maintained at 1.5 μg/mL, as determined by ICP-OES. Separately, 0.001 g of CNP-2 (detailed information is provided in the Supplementary Data) was dispersed in 10 mL of hexafluoroisopropanol via ultrasonication for 2 h, after which 3 g of Pluronic F-127 was added and stirred for another 2 h to yield Solution 2.

Using the Co-ESP setup (NANON-01A, MECC, Japan), Solution 1 and Solution 2 were simultaneously electrospun under 18 kV. The flow rates were set to 3 mL/h and 1 mL/h, respectively, with a 50 mm tip-to-collector distance, 25 mm/s nozzle movement speed, and a drum rotation speed of 800 rpm. After electrospinning 30 mL of Solution 1 and 10 mL of Solution 2, the composite fiber membrane was removed and designated as CBPP-1. To obtain CBPP-2, CBPP-3 and CBPP-4, the nanoparticle content in Solution 2 was adjusted to 0.01 g, 0.05 g, and 0.1 g, respectively, following the same procedure. Specific details regarding the characterization of CBPP’s physicochemical properties are provided in the Supplementary Data.

### Hemocompatibility assay

The rabbit red blood cells were washed three times with Phosphate-Buffered Saline (PBS) and diluted to 5% (v/v). Subsequently, 100 mg of CBPP 1-4 was mixed with 1 mL of the red blood cell suspension, incubated at 37°C, and then centrifuged at 1200 rpm for 5 min. Finally, 100 μL of the resulting supernatant was transferred to a 96-well plate, and its absorbance at 540 nm was measured using a Multiskan FC microplate reader (A_sample_). Red blood cells treated with 0.1% Triton X-100 and PBS were used as the positive control (A_triton_) and negative control (A_PBS_), respectively, whereas the absorbance of the material incubated with PBS alone was recorded as the material control (A_material_). Each sample was analyzed in triplicate, and the hemolysis ratio was calculated according to the following equation:


$$\text{Hemolysis}\;\text{ratio}\;(\%)=\frac{A_{\text{sample}-}A_{\text{material}}-A_{\text{PBS}}}{A_{\text{triton}}-A_{\text{PBS}}}\times 100\%$$

.

### Cytocompatibility

L929 mouse fibroblasts were used for the *in vitro* experiments and cultured at 37°C with 5% CO_2_ until sufficient cell numbers and normal morphology were obtained. According to ISO 10993-5, 0.5 g of each CBPP 1-4 sample was fully immersed in 5 mL of culture medium and incubated for 24 h at 37°C with 5% CO_2_. The mixture was then centrifuged at 1200 rpm for 3 min to collect the sample extract. L929 cells were seeded in 96-well plates at a density of 2 × 10³ cells/well, and after adhesion, the medium was replaced with the corresponding extract. Cell viability was evaluated on Days 1, 3 and 5 using the CCK-8 assay. Live/Dead staining was used to assess cell viability, while rhodamine-phalloidin and DAPI staining were performed to observe cell morphology.

### *In vitro* angiogenesis of HUVECs

The angiogenic capacity of CBPP-2 was assessed using rat bone marrow mesenchymal stem cells (HUVECs). After Matrigel-coated plates were incubated for 1 h, 1.5 × 10^6^ cells were seeded into each well and treated with the corresponding sample extract. Following 6 h of incubation, tube formation was observed and imaged using an inverted fluorescence microscope (Nikon ECLIPSE Ti, Japan).

### *In vitro* polarization of macrophages

RAW264.7 cells (5 × 10^4^) were seeded in 6-well plates and cultured with or without Lipopolysaccharides (LPS, 2 μg/mL) for 12 h, followed by treatment with sample extracts to assess macrophage polarization. After two days, cells and supernatants were collected by centrifugation. Cells were stained with anti-CD86 and anti-CD206, respectively. Cytokines associated with M1 (TNF-α, IL-1β) and M2 (TGF-β, IL-10) macrophages in the supernatants were measured using ELISA kits. In addition, RAW264.7 cells were fixed with 4% paraformaldehyde for 10 min, stained for CD86 and CD206, and observed under a fluorescence microscope.

### Antioxidant properties

The intracellular ROS-scavenging ability of CBPP-2 was assessed using a ROS assay kit (Solarbio®, China). L929 cells were seeded in 24-well plates at 5 × 10^4^ cells/well and cultured for 24 h. After stimulation with Rosup (2 μg/mL) for 2 h, the cells were washed three times with MEM and then incubated with fresh MEM containing 5% Fetal Bovine Serum (FBS) or CBPP-2 extract for 12 h. Finally, the cells were stained with 2′,7′-Dichlorofluorescin diacetate (DCFH-DA) or Dihydroethidium (DHE) in serum-free medium for 30 min and imaged using an inverted microscope (Nikon ECLIPSE Ti, Japan).

### Animal model and CBPP double-layer artificial dura mater implantation

Forty-eight male Sprague–Dawley rats were used for the *in vivo* study. After seven days of acclimation, the rats were randomly divided into four groups: Negative Control, Positive Control, CBPP-2 group (BG content: Si ion 1.5 μg/mL; CNP content: 0.1 wt%) and CBPP-0 group (BG content: Si ion 1.5 μg/mL; CNP content: 0 wt%). Samples were collected on postoperative Days 7, 14 and 28 for histological and biochemical analyses. All procedures were approved by the Ethics Committee of Sichuan Lilaisinuo Biological Technology Co., Ltd. (Accreditation No. LLSN-2024104). Detailed animal experiment procedures are provided in the Supplementary Materials. Pathological score are provided in the Supplementary Table S1.

### Statistical analysis

All quantitative data were expressed as mean ± standard deviation (SD), while qualitative or categorical data were presented as percentages or representative images. The normality of quantitative data was assessed using the Shapiro–Wilk test, and homogeneity of variance among groups was evaluated using Levene’s test. No heterogeneous variances were observed in the analyzed datasets. For normally distributed data (*P* > 0.05), one-way ANOVA followed by the Fisher’s Least Significant Difference (LSD) test was used for intergroup comparisons. For data that did not conform to a normal distribution (*P* < 0.05), the K-W method was first used to determine whether overall differences existed among multiple groups. When significant differences were detected, the M-W method was further applied for pairwise comparisons. All statistically significant differences are indicated with asterisks in the figures (*n* = 4, *P < 0.05, **P < 0.01, ***P < 0.001).

## Results and discussion

### Physicochemical properties of CBPP double-layer artificial dura mater

**Figure 1.**
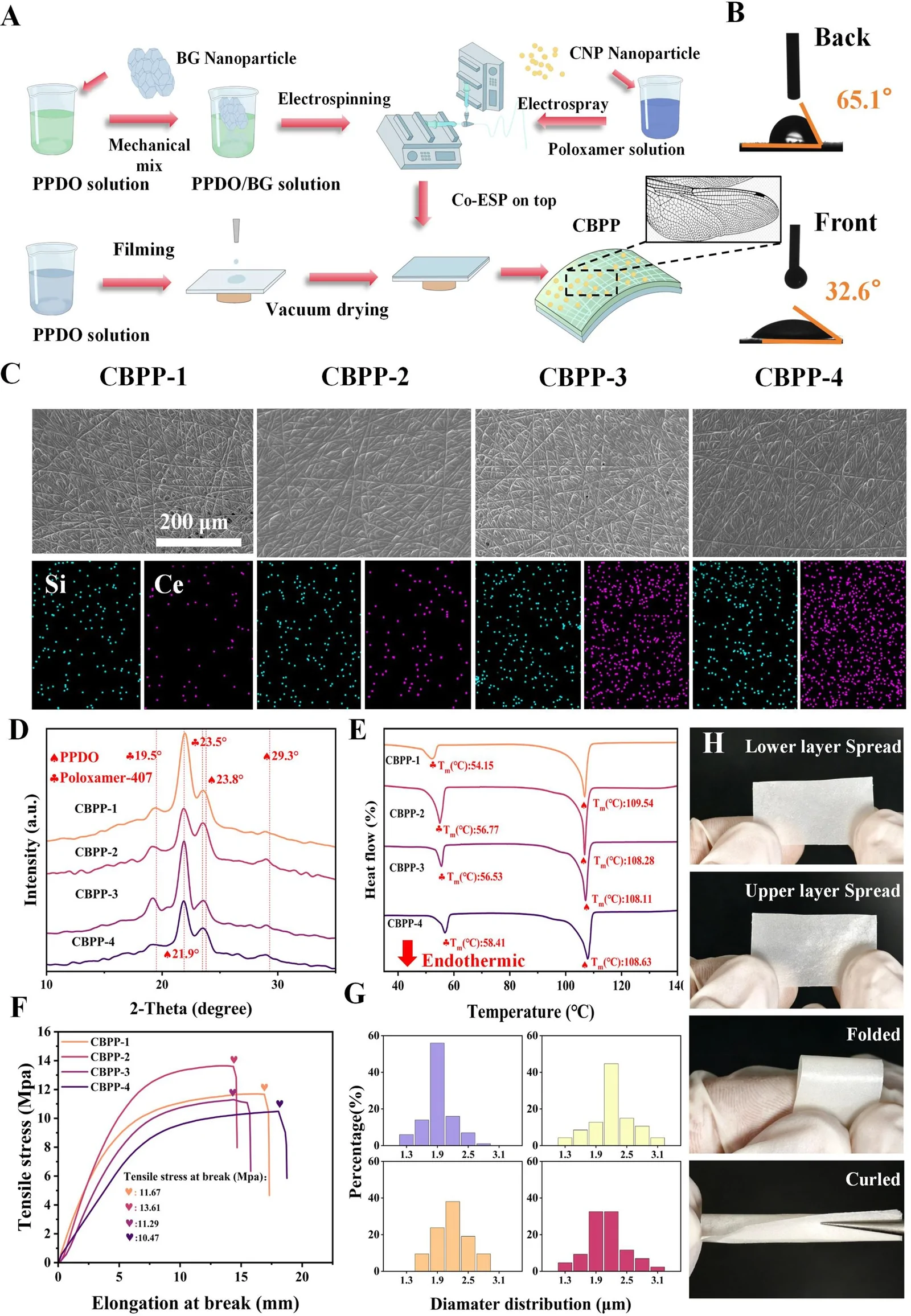
Physicochemical properties of CBPP 1-4. (**A**) Schematic illustration of fabrication process; (**B**) Water contact angle analysis; (**C**) SEM morphology and elemental mapping of CBPP; (**D–F**) XRD, DSC, and mechanical property analyses; (**G**) Diameter distribution; (**H**) Photographs to demonstrate the flexibility of CBPP.

The physicochemical properties of BG and CNP are characterized in Supplementary Figure S3. Following the Co-ESP process combining Pluronic F-127-loaded CNP with BG-containing PPDO, a bilayer fibrous membrane was successfully fabricated and subjected to a series of physicochemical characterizations. As shown in Figure 1B, the pure PPDO layer on the membrane’s backside exhibited a water contact angle of 65.1°, consistent with previous studies. The front surface showed a significantly lower contact angle of 32.6°, attributed to the hydrophilic functional groups introduced by F-127. This dual hydrophilic structure is advantageous for dura repair. Owing to its excellent thermoresponsive behavior, the F-127 layer can undergo effective gelation under physiological temperature conditions, thereby exhibiting considerable potential to prevent cerebrospinal fluid leakage [45]. Meanwhile, the inward contraction of hydrophilic groups after gelation is expected to significantly reduce adhesion between artificial dura mater and brain tissue while releasing CNP slowly. Figure 1C presents the surface morphology and elemental distribution of the membrane. Dragonfly wings are mainly composed of longitudinal/cross veins and thin membranes, forming a lightweight hierarchical nanocomposite structure that provides high load-bearing capacity, flexibility, and structural stability during flapping, gliding, and hovering flight [48, 49]. In particular, the vein network functions as a reinforcing skeleton, whereas the membrane contributes to continuous surface coverage and stress transfer. This natural architecture offers an instructive model for dural substitute design, in which the electrospun PPDO/BG fibrous network mimics the vein-like reinforcing framework, while the F-127/CNP phase distributed among the fibers resembles the membrane-like interfacial matrix. Such a vein-membrane-inspired microstructure is expected to simultaneously provide mechanical support, interfacial integrity, and a bioactive microenvironment for cell adhesion and tissue regeneration. Elemental mapping revealed a small but uniform presence of Si, confirming evenly dispersed BG across all groups, while the Ce signal intensity increased progressively with higher nanoparticle content, corresponding to the doping gradient. The morphology closely resembled the microstructure of a dragonfly wing, and together with the elemental analysis, confirmed the successful fabrication of the bioinspired dura mater membrane. Figure 1G displays the fiber diameter distribution of CBPP. The ‘main vein’ of PPDO-based dragonfly wings shows minimal variation in diameter, all remaining around 2 μm, while the poloxamer-based ‘wing membrane’ structure is uniformly distributed between the veins. This biomimetic structure demonstrates excellent degradation cycle control. Figure 1H further highlights CBPP’s outstanding flexibility and foldability, making it suitable for dural repair and facilitating surgical procedures.

Physicochemical evaluations were performed on the membrane’s functional surface (Figure 1D and E). Both X-Ray Diffraction (XRD) and Differential Scanning Calorimetry (DSC) analyses exhibited characteristic peaks corresponding to the two main components. XRD patterns showed diffraction peaks for F-127 at 19.5° and 23.5°, and for PPDO at 21.9°, 23.8° and 29.3°. In the DSC curves, thermal melting peaks were observed at approximately 55°C and 110°C, corresponding to F-127 and PPDO, respectively, confirming successful composite formation. Tensile testing revealed a maximum tensile strength exceeding 10 MPa and an elongation at break of approximately 15 mm, demonstrating a favorable balance of stiffness and flexibility that meets both the mechanical requirements of dura mater and the handling demands of surgical applications. Following successful fabrication, subsequent cellular experiments will be conducted to evaluate the biocompatibility and functional performance of CBPP double-layer artificial dura mater.

### *In vitro* degradation and ions release capacity

**Figure 2.**
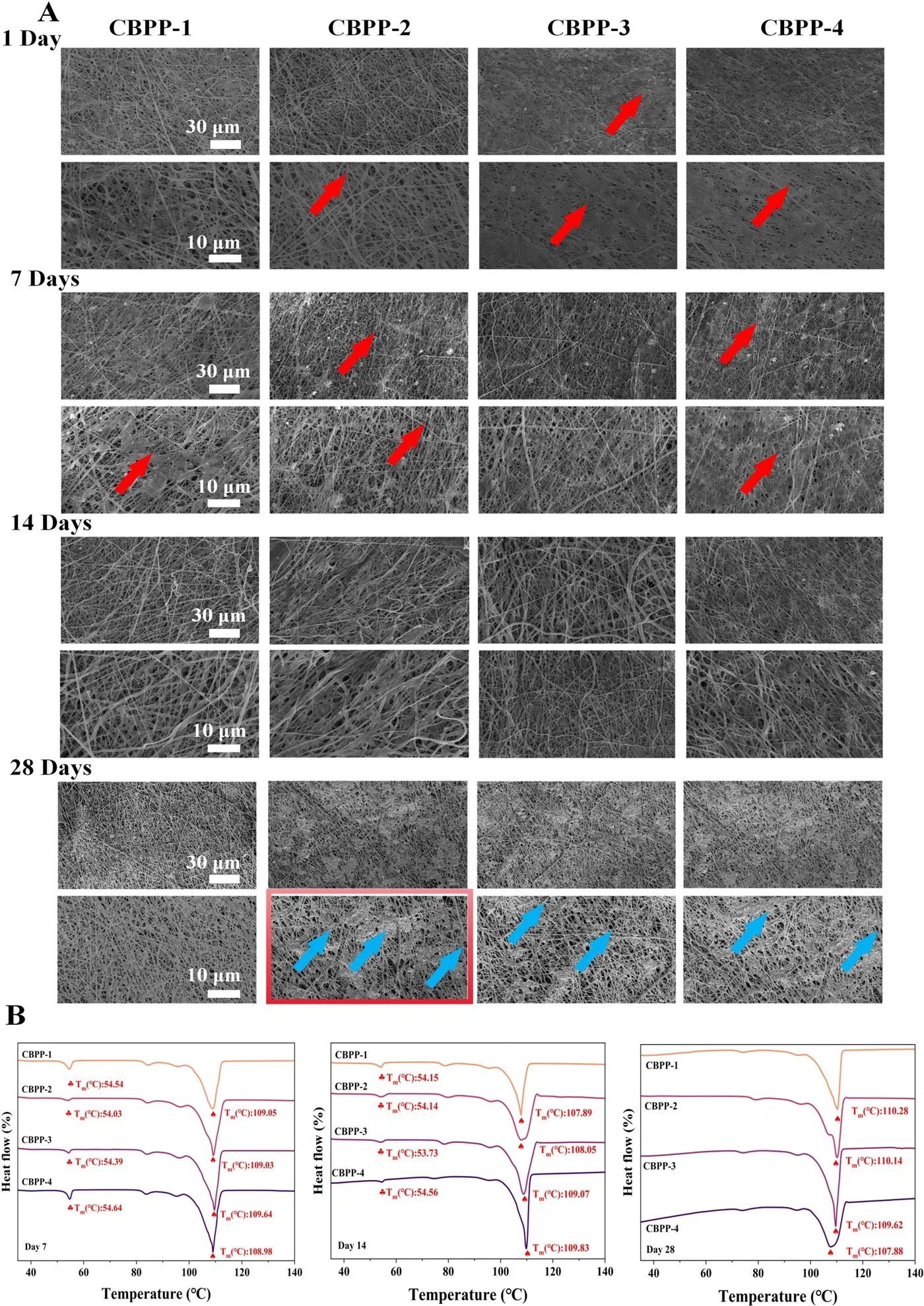
*In vitro* degradation behavior of CBPP 1-4. (**A**) Morphological evolution during the *in vitro* degradation; (**B**) DSC changes after 7, 14, and 28 days. (Blue arrow: bioactive compounds; Red arrow: residual F-127.)

**Figure 3.**
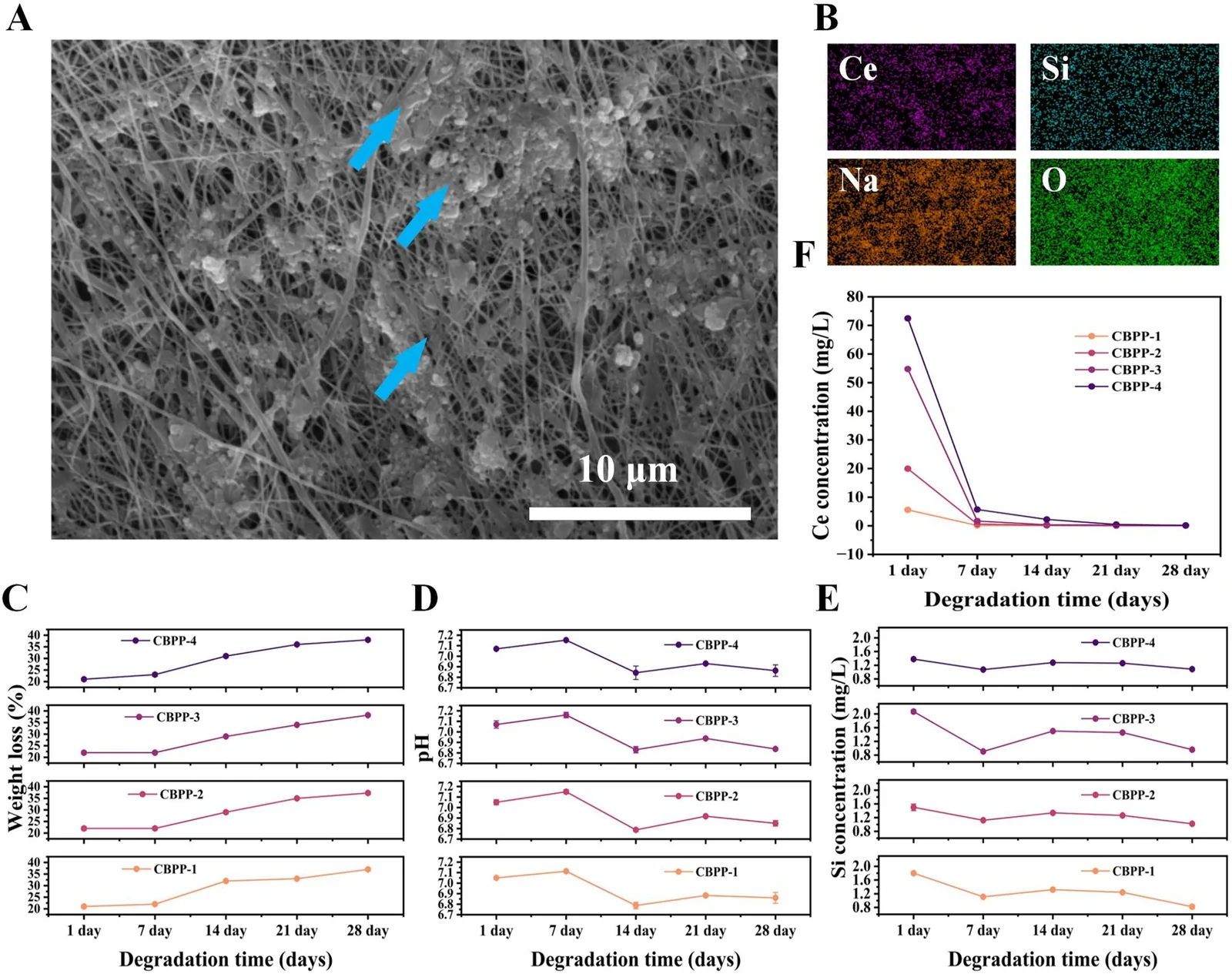
*In vitro* detailed ion-controlled release capability of CBPP 1-4. (**A**–**B**) Morphology and elemental analysis of samples after 28 days of *in vitro* mineralization; (**C–F**) Mass loss, pH variation, and sustained release behavior throughout the degradation period. (Arrow: bioactive compounds.)

Figure 2A illustrates the morphological changes of CBPP 1-4 during *in vitro* degradation. Within the first day, the F-127 hydrogel in the electrospun PPDO fiber matrix partially dissolved, revealing the characteristic electrospun fibrous structure. A small amount of residual hydrogel remained attached to the fiber surface. By Day 7, further degradation of the Pluronic component was evident, and DSC analysis (Figure 2B) confirmed the presence of faint Pluronic peaks. Between Days 7 and 14, this trend intensified, accompanied by the collapse of the fibrous network. Fibers became curved and softened, while the embedded BG began to release gradually. As shown in Figure 2B, the DSC peaks corresponding to Pluronic F-127 became nearly undetectable, and by Day 28, DSC results revealed the complete disappearance of the hydrogel phase. At this stage, the surface exhibited distinct mineralization characteristics of BG, indicating excellent bioactivity of the membrane. Elemental analysis (Figure 3B) suggested the presence of cerium oxides combined with sodium-calcium silicates.

Figure 3C presents the mass loss profiles of the CBPP groups. On Day 1, the mass loss of all CBPP samples reached approximately 20%, which was primarily attributed to the dissolution of F-127. Between Days 1 and 7, the degradation rate became relatively slow, likely because PPDO underwent gradual hydrolysis after the complete dissolution of F-127. This behavior is consistent with the intended design strategy, whereby the bioactive ions loaded within the PPDO matrix are released in a sustained manner during this stage to promote angiogenesis. After Day 7, the degradation rate increased significantly. Correlating SEM observations with DSC results, the degradation process can be divided into two phases: detachment of most of the surface-bound F-127, followed by progressive structural collapse of the PPDO fiber matrix itself. By Day 28, the total mass loss reached approximately 45%. This degradation timeline ensures a graded and controlled release of functional components within the desired repair period, promoting cellular growth during regeneration while allowing complete biodegradation into non-toxic byproducts afterward. Soft tissue healing is generally divided into three sequential stages: inflammation (Days 0–4), angiogenesis (Days 4–14), and extracellular matrix remodeling with collagen deposition (Days 14–28). In our design, the release profiles of CNP from the F-127 and BG-derived bioactive ions from the PPDO fibers were deliberately tailored to coincide with these physiological healing stages.

The pH variation and ion release data further confirmed this staged degradation behavior. As shown in Figure 3D, during the first week, the slight rise in pH was attributed to the dissolution of surface BG, releasing alkaline ions into the medium. This phenomenon is consistent with the Si ion release profiles observed in Figure 3E. Previous studies have reported that Si concentrations in the range of approximately 0.5–3 mg/L exhibit the most effective pro-angiogenic activity [50]. As shown in Figure 3E, the sustained Si ion release profiles of the samples consistently remained within this optimal range. Therefore, CBPP-2 can be considered to possess excellent sustained Si ion release capability, endowing it with the potential to stimulate angiogenesis and thereby accelerate the repair process. Figure 3F presents the Ce release profile. The Ce concentration reached its maximum on Day 1, followed by a sharp decline beginning on Day 7. This phenomenon can be primarily attributed to the rapid dissolution of F-127 in aqueous environments. By Day 14, the Ce concentration had become nearly undetectable. Between Days 7 and 14, the PPDO membrane began to collapse. Reported studies indicate that PPDO degradation products are mildly acidic, lowering the pH to around 5, whereas the dissolution of BG releases alkaline ions, counterbalancing the acidity and stabilizing the pH near 6.8. Maintaining a neutral pH facilitates wound healing [51]. From Days 14 to 21, this dynamic equilibrium persisted, maintaining the pH close to 7.0 as both PPDO and BG degraded simultaneously. By Days 21–28, the mineralization of BG was complete, and PPDO degradation became dominant, resulting in a slight decrease of pH back to 6.8.

Overall, the degradation process closely aligns with the natural phases of wound healing: during Days 0–7, CNP released from the F-127 hydrogel regulates the inflammatory response. From Day 7, BG released from the PPDO matrix accelerates tissue repair. After Day 21, the remaining fibrous supports neotissue formation following mineralization. Collectively, these findings confirm the successful fabrication of the intended bioinspired, temporally degradable artificial dura mater.

### Cytotoxicity, angiogenesis and reactive oxygen species clearance ability of CBPP 1-4 double-layer artificial dura mater

**Figure 4.**
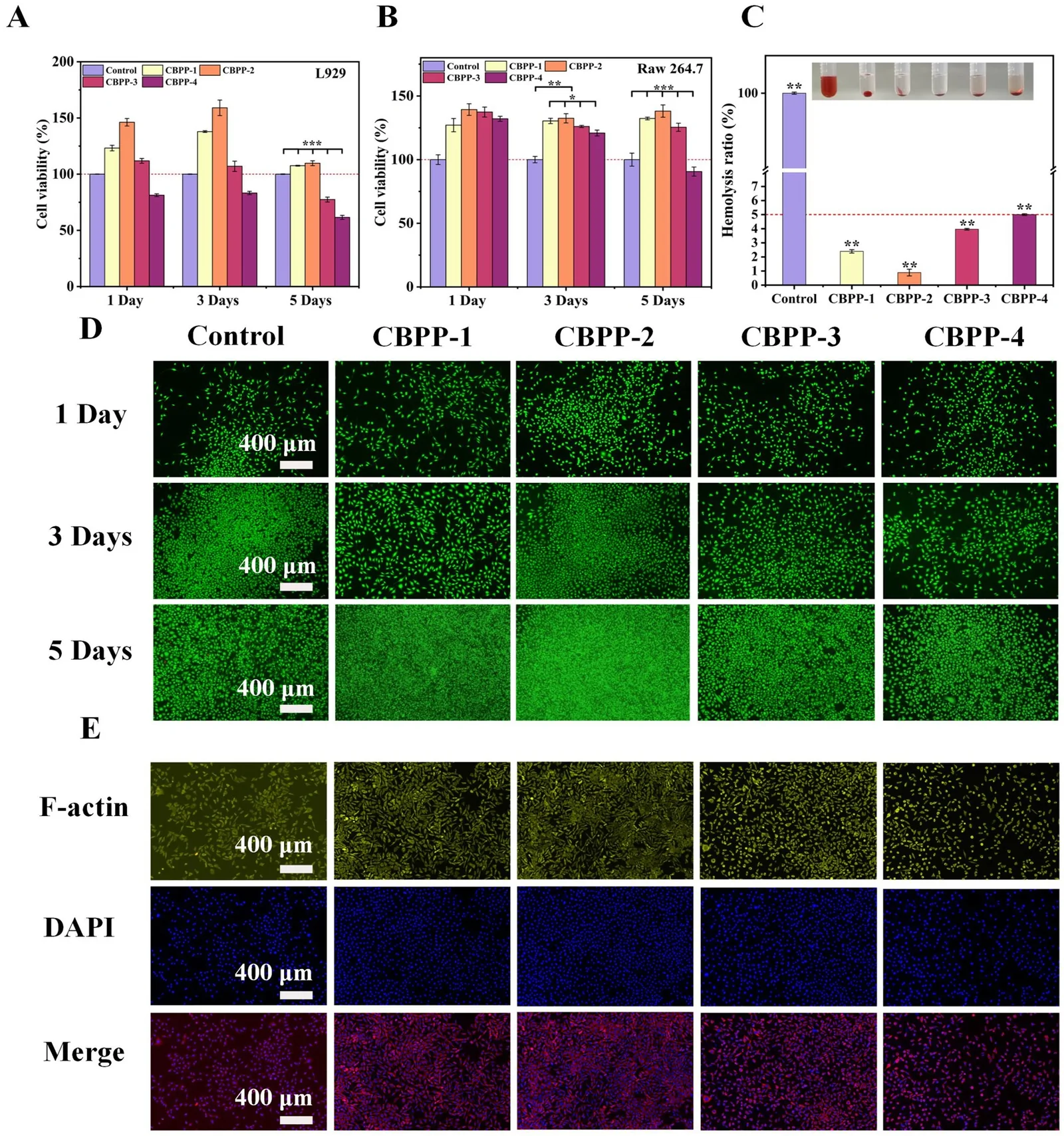
Cellular compatibility and blood compatibility of CBPP1-4. (**A–B**) CCK-8 cytotoxicity evaluation of CBPP; (**C**) Hemolysis assay results of CBPP; (**D–E**) live/dead staining and cytoskeleton/nucleus fluorescence double staining of CBPP.

As shown in Figure 4A and B, the cytotoxicity of the artificial dura mater was evaluated using L929 and RAW 264.7 cell lines. The CCK-8 results revealed that for L929 cells, both CBPP-4 groups exhibited proliferation rates below 80% after five days, while CBPP-3 also showed cytotoxicity on Day 5. In contrast, CBPP-1 and CBPP-2 maintained proliferation rates exceeding 100% throughout the five-day period, likely due to the moderate presence of CNP, which can scavenge ROS and promote cell proliferation. However, excessive CNP concentrations may trigger the release of lactate dehydrogenase (LDH) and tumor necrosis factor-α (TNF-α), leading to apoptosis [52, 53].

A similar trend was observed in RAW 264.7 cells. However, all four groups maintained proliferation rates above 80%, indicating no evident cytotoxicity. Significant differences emerged among the groups by Day 3, suggesting that the addition of CNP positively influenced macrophage proliferation. By Day 5, CBPP-4 showed a decrease in proliferation relative to the control, likely due to excessive LDH and TNF-α release induced by high CNP content. The discrepancy between L929 and RAW 264.7 responses may result from macrophage polarization: ROS scavenging by CNP likely promoted M2-type polarization in RAW 264.7 cells, which secrete growth factors that enhance cell proliferation. Nevertheless, at later stages, excessive CNP concentration induced apoptosis-related factors, again reducing proliferation.

Hemolysis and fluorescence staining assays further verified the biocompatibility of CBPP 1-4. As shown in Figure 4C, mild hemolysis was observed in CBPP-4, whereas all other groups exhibited hemolysis rates below 5%. Live/dead staining of L929 cells (Figure 4D) correlated well with the CCK-8 results. Notably, the CBPP-1 and CBPP-2 groups displayed markedly higher cell density and confluence than the others by Day 5, confirming that appropriate CNP incorporation enhances proliferation. Cytoskeletal staining (Figure 4E) revealed that except for CBPP-4, cells in all groups maintained normal morphology, with clearly defined nuclei and cytoplasm, typically exhibiting spindle-shaped fibroblast forms. In contrast, CBPP-4 cells displayed nuclear condensation, cytoplasmic shrinkage, and partial rounding, indicative of apoptotic stress.

Collectively, these findings demonstrate that excessive CNP doping induces cytotoxicity, whereas moderate incorporation exerts a beneficial regulatory effect on cell proliferation. Considering both biocompatibility and performance, CBPP-2 was selected for subsequent experiments.

**Figure 5.**
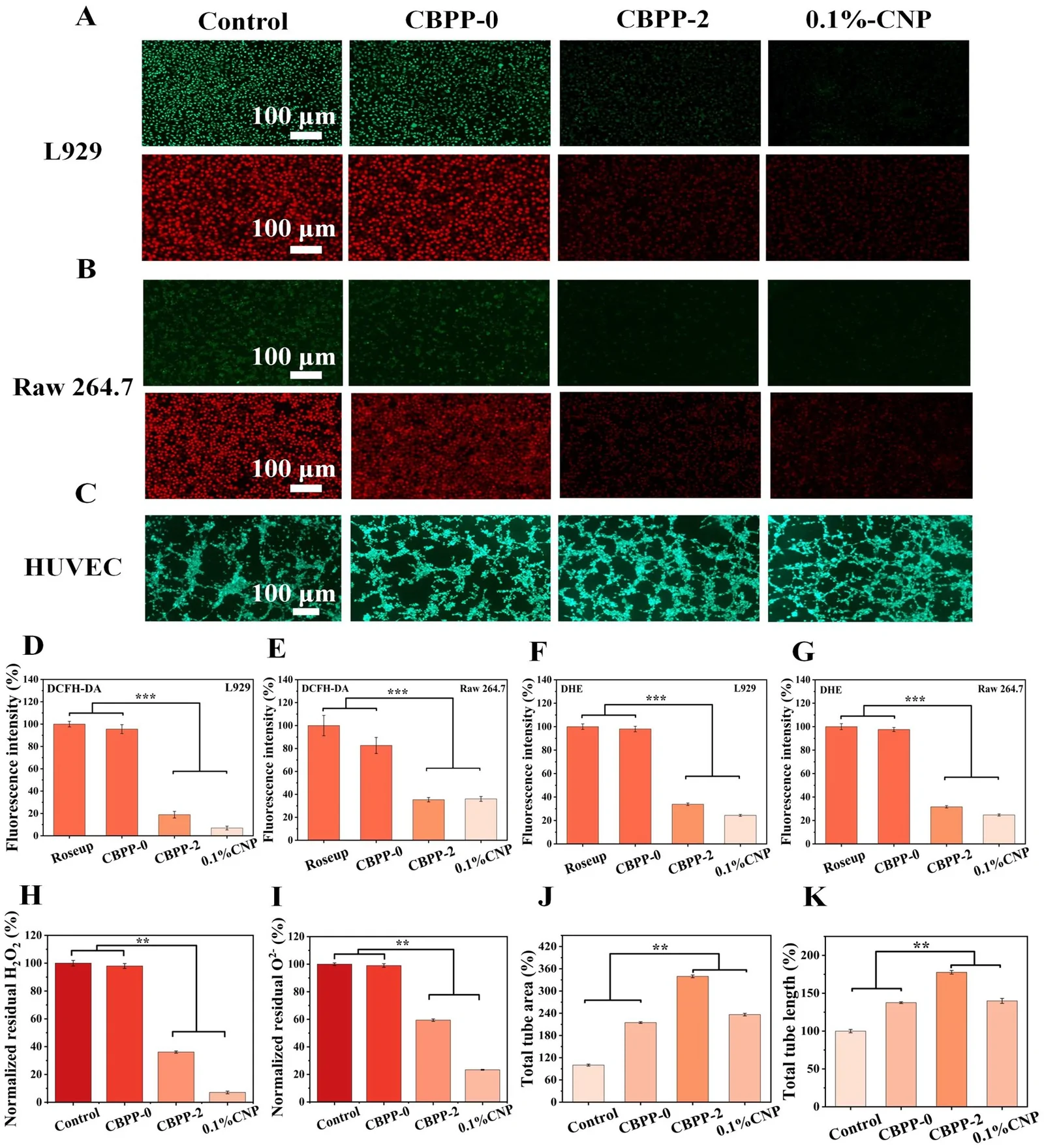
*In vitro* ROS scavenging and angiogenic capacity of CBPP-2. (**A**) DCFH-DA and DHE fluorescence staining showing ROS scavenging capability of CBPP-2 in L929 cells; (**B**) DCFH-DA and DHE fluorescence staining of CBPP-2 in RAW 264.7 cells; (**C**) *In vitro* angiogenic activity of HUVECs; (**D–E**) Quantitative fluorescence analysis of L929 cells; (**F–G**) quantitative fluorescence analysis of RAW 264.7 cells; (**H–I**) *In vitro* hydrogen peroxide and superoxide anion scavenging assays; (**J–K**) Quantitative analysis of HUVEC angiogenesis *in vitro*.

The antioxidant capacity of the artificial dura mater was evaluated using a cellular oxidative stress model. As shown in Figure 5A and B, the artificial membranes exhibited similar ROS-scavenging behavior in RAW 264.7 and L929 cells. Within the same field of view, the CBPP-2 and CNP groups displayed markedly lower fluorescence intensities of DCFH-DA and DHE staining compared with the other groups. Quantitative fluorescence analysis (Figure 5D–G) confirmed a highly significant reduction in intracellular reactive oxygen species (ROS) levels in the CBPP-2 and CNP groups relative to CBPP-0 and the Control. Furthermore, hydrogen peroxide and superoxide anion scavenging assays (Figure 5H and I) produced consistent results, collectively demonstrating that CNP-loaded artificial dura mater possesses excellent ROS-scavenging capability *in vitro*.

Figure 5C, J, and K present the fluorescence staining and quantitative analysis of angiogenesis for CBPP-2. The results showed that CBPP-2, incorporating both CNP and BG, exhibited a markedly superior angiogenic capacity compared with the other groups, with statistically significant differences between CBPP-2/CNP and CBPP-0/Control groups. Previous studies have shown that BG promotes angiogenesis through the release of Si ions, which stimulate vascular endothelial growth factor (VEGF) secretion, whereas CNP modulates oxidative stress and induces macrophage polarization, leading to the secretion of growth factors such as TGF-β that further enhance vascular formation [28, 29]. These findings indicate that the combined incorporation of BG and CNP achieves a more potent pro-angiogenic effect than conventional single-component approaches.

### *In vitro* immunomodulatory effect of CBPP double-layer artificial dura mater

**Figure 6.**
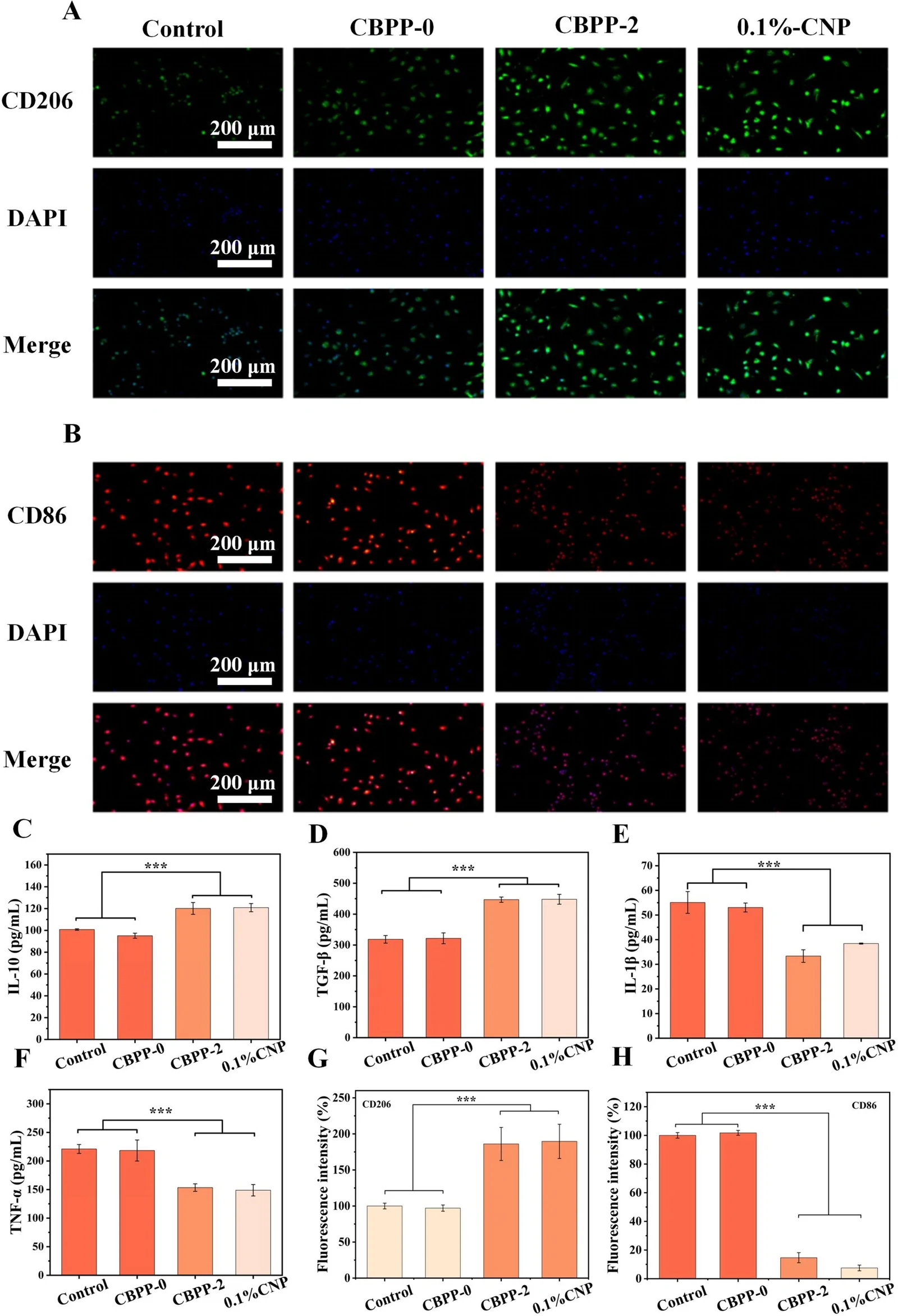
*In vitro* polarization of macrophages by CBPP-2. (**A–B**) Fluorescence staining of macrophage polarization; (**C–F**) secretion levels of IL-10, TGF-β, IL-1β and TNF-α in macrophage suspensions; (**G–H**) Quantitative analysis of macrophage polarization fluorescence staining.

Macrophages play an important role in both the initiation and maintenance phases of tissue repair, particularly through their regulation of phenotypic polarization. M1 macrophages exhibit pro-inflammatory characteristics, whereas M2 macrophages suppress inflammation and promote tissue regeneration. As shown in Figure 6A and B, compared with the CNP group, the Control group displayed a marked increase in CD86 expression (M1 macrophage marker) and a decrease in CD206 expression (M2 macrophage marker), indicating that LPS stimulation enhanced M1 polarization while inhibiting M2 polarization. In contrast, the CBPP-2 group showed a significant reduction in CD86 levels and a pronounced increase in CD206 expression relative to the Control, with fluorescence quantification confirming statistically significant differences (Figure 6G and H). These findings demonstrate that CBPP-2 effectively suppressed M1 polarization while promoting M2 polarization in macrophages.

The secretion levels of pro-inflammatory cytokines (TNF-α and IL-1β) and anti-inflammatory cytokines (TGF-β and IL-10) were assessed to evaluate the immunoregulatory effect of CBPP-2 (Figure 6C–F). The results revealed that TNF-α and IL-1β levels were significantly reduced in the CBPP-2 group compared with the Control, whereas TGF-β and IL-10 secretion markedly increased, confirming the strong anti-inflammatory and immunomodulatory potential of the CBPP-2 double-layer artificial dura mater. These results are consistent with the findings reported by Du *et al.* [29]. They further demonstrated that conditioned medium derived from M2-polarized macrophages exhibited superior pro-angiogenic effects in angiogenesis assays. In this study, the hierarchically degradable structure of the artificial dura mater synergistically integrates these mechanisms, resulting in a pronounced enhancement of angiogenic activity.

### *In vivo* dura mater defect repair efficacy of the CBPP-2

**Figure 7.**
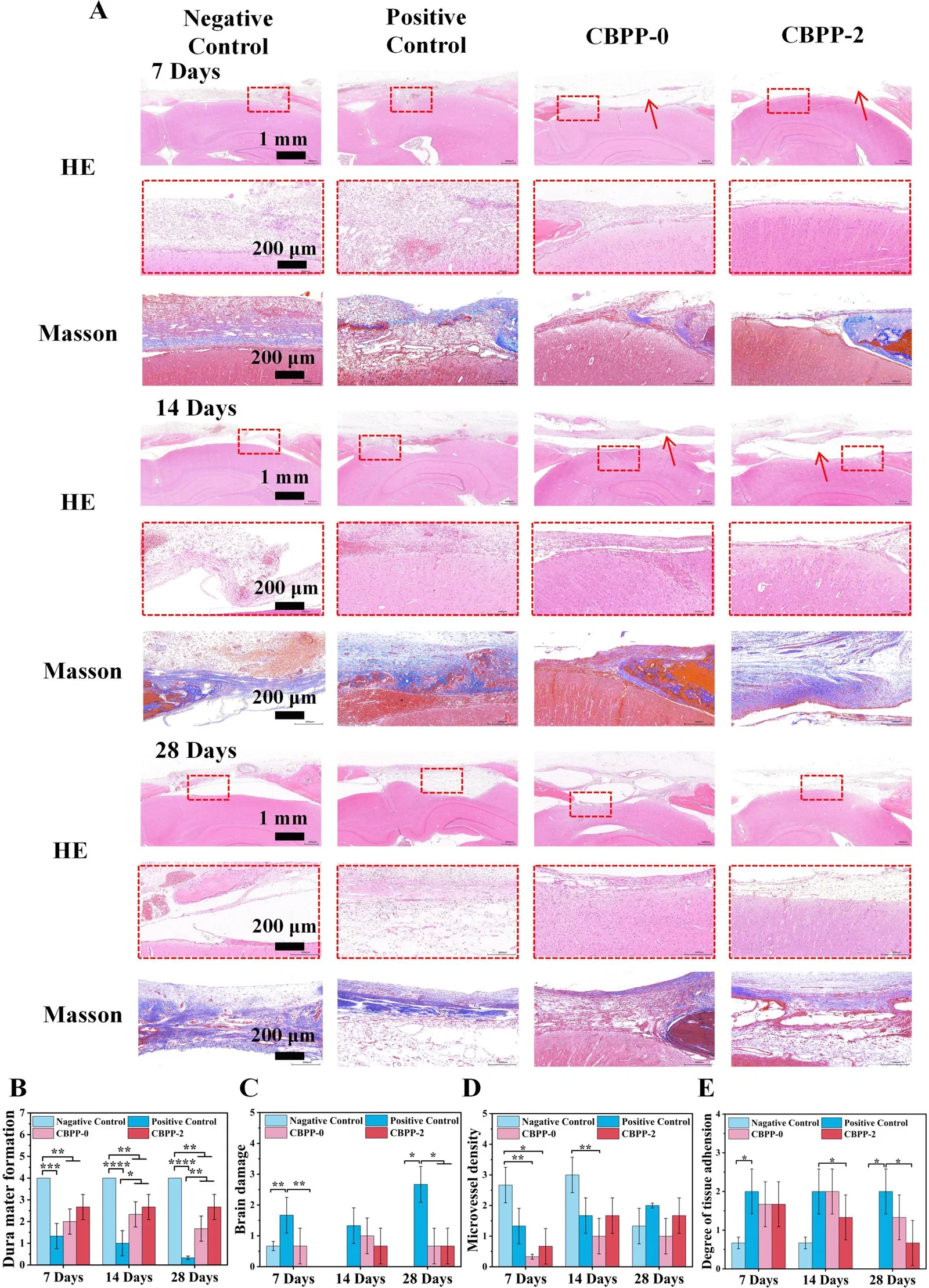
The repair efficacy of CBPP-2 on rat dural defect. (**A**) Representative Hematoxylin and Eosin (H&E) and Masson staining images; (**B–E**) Quantitative analyses of dura mater formation, neovascularization, brain damage, and adhesion degree. (Arrow: location of the implanted material.)

All animals survived after implantation, with no signs of infection or severe foreign body reaction observed (Supplementary Figure S2). As shown in Figure 7A, at the same postoperative time points, the Negative Control group exhibited intact brain tissue morphology with a clearly distinguishable dura mater. Fibrous tissue was observed at the cranial defect site, with only mild adhesion between the fibrous layer and brain tissue, and no signs of neural injury. In contrast, the Positive Control group showed extensive fibroblast proliferation at the defect site, accompanied by minor hemorrhage and inflammatory cell infiltration, with evident adhesion between brain tissue and proliferating fibers (Figure 7E). Moreover, the dura structure in this group was indistinct due to tissue damage and deep fibroblast ingrowth into the injured area. In both CBPP-0 and CBPP-2 groups, a thin fibrous connective layer covered the original dural site with only slight adhesion to brain tissue, showing markedly reduced injury compared to the Positive Control group (Figure 7D). Mao *et al.* [41] reported partial adhesion formation in rat models treated with PPDO fibrous membranes alone. The absence of this phenomenon in the CBPP groups can be attributed to the contraction of the hydrophilic end within the F-127 hydrogel, which reduced interfacial adhesion between the artificial dura mater and the surrounding tissues [43]. Neovascularization was observed at all three time points (Figure 7B), with the CBPP-2 group demonstrating significantly enhanced angiogenesis compared with CBPP-0, likely due to the synergistic effect of CNP and BG in promoting vascular formation. Both CBPP-0 and CBPP-2 regenerated dura exhibited more complete and continuous structural integrity than the Positive Control group, with statistically significant differences at all time points. Masson staining revealed that by postoperative Day 14, CBPP-2 had already formed a continuous, structurally complete dura layer with uniform collagen distribution (Figure 7B). This improvement can be attributed to the presence of CNP, which mitigated inflammation, shortened the inflammatory phase, and accelerated organized tissue regeneration, demonstrating superior biocompatibility and regenerative performance of the CBPP-2 hydrogel fibrous. Correlating these results with the degradation and release profiles, CBPP-2 exhibited a rapid release of CNP within the first 7 days, followed by a sustained release of BG up to 28 days, aligning well with the critical phases of tissue repair. This sequential release pattern effectively harnessed the early anti-inflammatory role of CNP and the prolonged regenerative stimulation of BG, enhancing angiogenesis and collagen deposition. Immunofluorescence staining further confirmed that CBPP-2 significantly increased CD206-positive M2 macrophages at postoperative Day 7 (Figure 7C and D), indicating effective polarization toward an anti-inflammatory phenotype. This finding was consistent with the *in vitro* macrophage polarization results, suggesting that M2 macrophages established a favorable immunoregulatory microenvironment conducive to tissue repair.

**Figure 8.**
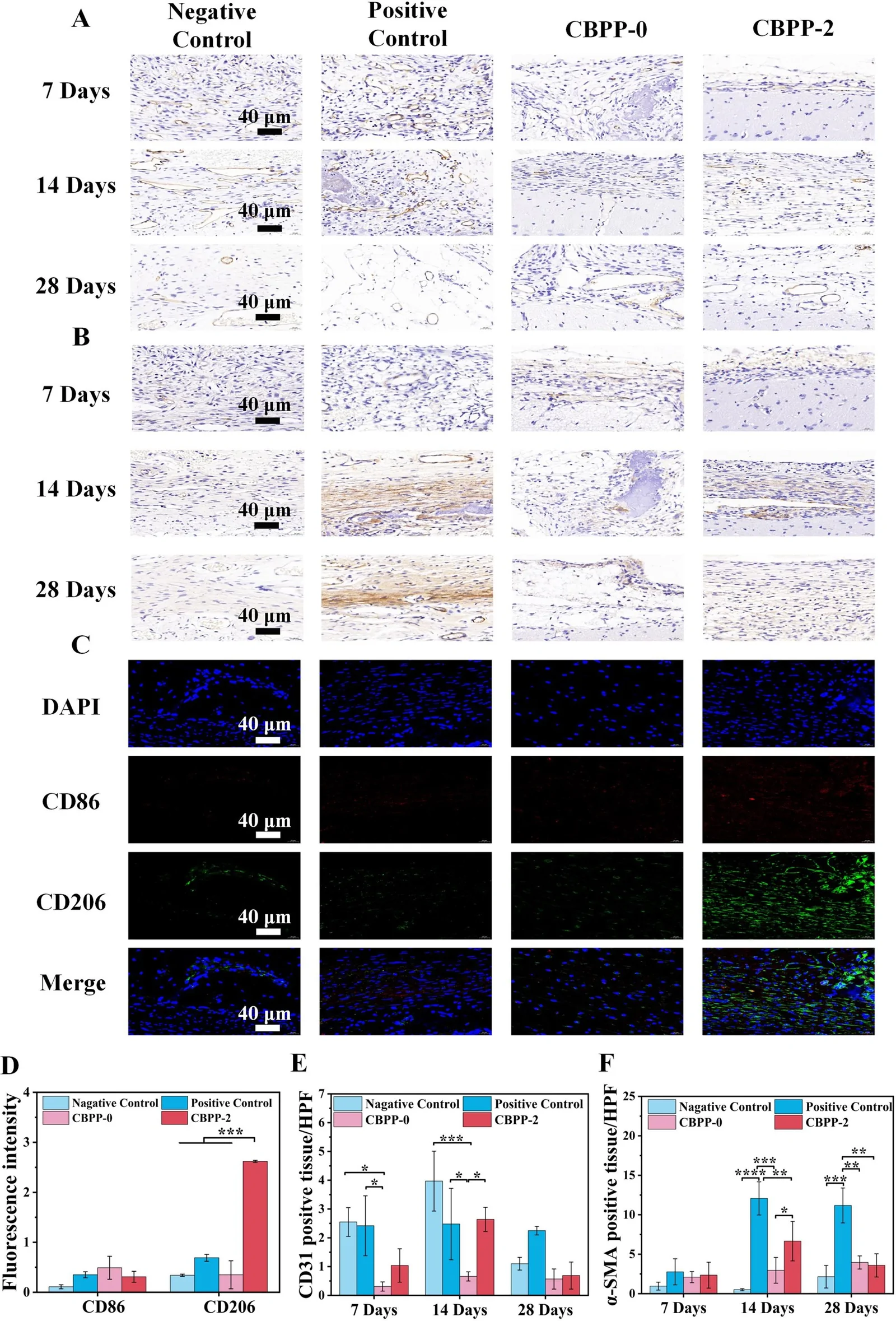
Immunohistochemical and immunofluorescence staining of the rat dural defect model. (**A–B**) Immunohistochemical staining of CD31 and α-SMA; (**C**) Dual immunofluorescence staining of CD86 (red) and CD206 (green) after seven days of implantation; (**D–F**) Quantitative analyses of immunofluorescence and immunohistochemical results.

CD31 immunohistochemistry (Figure 8A) revealed that CBPP-2 induced significantly greater neovascular formation at Day 14 than CBPP-0 (Figure 8E). However, the Positive Control group exhibited the highest microvessel density among all groups, likely due to excessive inflammatory stimulation and pathological hyperplasia following tissue injury. This phenomenon was further clarified through α-SMA staining (Figure 8B), which showed abundant disorganized myofibroblast proliferation in the Positive Control group, indicating fibrotic overgrowth and structural disarray. In contrast, the CBPP-2 group displayed α-SMA-positive regions aligned along the direction of fibrous tissue, suggesting well-organized tissue regeneration. No apparent myofibroblast aggregation was observed, indicating that CBPP-2 guided a normal repair process with minimal abnormal proliferation. At Day 14, both α-SMA and CD31 expressions showed significant differences between the CBPP-2 and CBPP-0 groups (Figure 8E and F), with CBPP-2 exhibiting normal protein morphology and organized vascular patterns, distinct from the excessive fibrosis observed in the Positive Control group.

By Day 28, CBPP-2 had formed a continuous, well-aligned neodura with orderly collagen architecture and a clear boundary with surrounding native tissue (Figure 8B). Combined with previous findings, these results indicate that the CBPP-2 hydrogel fibrous artificial dura mater achieved temporally controlled release of CNP and BG, coordinating inflammation modulation, angiogenesis, and tissue regeneration while effectively preventing postoperative adhesion. The degradation profile of CBPP-2 closely matched the natural timeline of tissue repair, providing a biomaterial that synchronizes inflammatory regulation and regenerative remodeling. Altogether, the CBPP-2 presents a bioinspired, time-programmed regenerative strategy that offers new insights into the mechanisms of dural repair and holds great promise for clinical applications in dural tissue reconstruction.

## Conclusion

In this study, we developed a bioinspired double-layer artificial dura mater that exhibited excellent mechanical strength, biocompatibility, and a time-programmed degradation profile matching the natural dural repair cycle. The temperature-sensitive properties of F-127 significantly reduced adhesion between the artificial dura mater and tissues. CNP effectively reduced oxidative stress and modulated macrophage polarization toward the M2 phenotype, while BG promoted angiogenesis and collagen deposition. Their synergistic release enabled sequential inflammation control and tissue regeneration. *In vivo* experiments confirmed that CBPP-2 facilitated complete neodura formation in 14 days and reduced fibrosis and adhesion at the same time. However, long-term evaluation and large-animal validation are still needed. Meanwhile, the present study did not investigate the underlying mechanisms at the molecular level, such as gene signaling pathways, which represents a certain limitation of this work. Overall, this work presents a bioinspired, temporally responsive dura mater scaffold that couples mechanical protection with immunomodulation and angiogenesis, offering an excellent strategy for advanced dural repair and biomaterial design.

## Supplementary Material

rbag154_Supplementary_Data
