# Supplementary material for: Bioinspired BG/PPDO-CNP/Pluronic F-127 hydrogel fibrous dura mater with temporally programmed degradation and adhesion prevention inspired by the hierarchical architecture of dragonfly wings
Source: Regen Biomater. 2026 Jul 22;13:rbag154. doi: 10.1093/rb/rbag154 (PMC13616049; doi:10.1093/rb/rbag154)
Supplement: Supplementary file 1 [file rbag154_supplementary_data.zip › Supporting Information.docx]

Supporting Information

**Bioinspired BG/PPDO-CNP/Pluronic F-127 hydrogel fibrous dura mater with temporally programmed degradation and adhesion prevention inspired by the hierarchical architecture of dragonfly wings**

*Junqin Mao^a,#^, Heng Zheng^a,#^, Qiuyu Zeng^a^, Wanli Zhang^a^, Qiang Liu^a^, Tao Shen^a^, Xinyu Zhang^a^, You Tang^a^, Wei Tang^a^, Guoyu Lv**^a,[1]*^*

**Materials information**

PPDO was purchased from Evonik Industries. 45S5 bioactive glass was was purchased from Jiangsu Qiuzheng New Material Technology Co., Ltd.. Hexafluoroisopropanol ((CF₃)₂CHOH, ≥99.0%), cerium(III) nitrate hexahydrate (Ce(NO_3_)_3_·6H_2_O, ≥99.0%), oleylamine (C_18_H_37_N, ≥99.0%), and 1-octadecene (C_18_H_36_, ≥99.0%) were obtained from Aladdin Inc. (Shanghai, China).

**Scanning electron microscopy (SEM)**

The morphological features and microstructures of CBPP 1-4 were characterized using an Oxford Nova NanoSEM450 scanning electron microscope. The elemental distribution on the sample surfaces was further assessed by energy dispersive spectroscopy (EDS).

**Transmission electron microscope (TEM)**

CNPs and BG particles were first dispersed in anhydrous ethanol, then deposited onto copper grids by drop casting and subsequently observed using TEM.

**X-ray diffraction (XRD) and X-ray photoelectron spectroscopy (XPS)**

Uniformly dispersed CNPs in anhydrous ethanol were freeze-dried under vacuum for 12 h to obtain CNP powders. X-ray diffraction (XRD) analysis of CNP powders and CBPP 1-4 samples was performed within the 2θ range of 5°-80°. X-ray photoelectron spectroscopy (XPS) measurements were conducted on CNP powders, with spectra calibrated using the C 1s peak at 285 eV for survey scans and 284.6 eV for high-resolution spectra.

**Water contact angle (WCA)**

The water contact angles on both surfaces of CBPP 1-4 were measured using an optical contact angle meter (KRÜSS DSA25, Germany).

**Differential scanning calorimeter (DSC)**

Briefly, 50 mg of each CBPP 1-4 sample was cooled to -20 °C at a rate of 5 °C/min, maintained for 5 min to eliminate previous thermal history, and subsequently heated to 150 °C at a rate of 5 °C/min.

**Tensile properties**

The tensile performance of the membrane samples was assessed using an electronic universal testing machine (INSTRON, USA). Tensile tests were performed on standard rectangular specimens prepared according to Type 2 of ISO 527-3, with a length of 150 mm, width of 20 mm, gauge length of 50 mm, and thickness of 0.15 mm.

***In vitro* Degradation properties of CBPP-2**

Briefly, 500 mg of each CBPP-2 sample was immersed in 5 mL of PBS and incubated in a constant-temperature shaker at 37 ± 1 °C for 1, 7, 14, 21, and 28 days, with PBS replaced every 3 days. At each time point, degraded samples and corresponding PBS solutions were collected for further analysis.

The pH of the PBS solution was measured using a digital pH meter (PHS-25, LeiCi, China). Si and Ce ion concentrations were determined by inductively coupled plasma optical emission spectrometry (Agilent 5100 SVDV, USA). For weight-loss analysis, samples with an initial dry weight of 500 mg were retrieved, rinsed with deionized water, and vacuum-dried at 40 °C for 24 h to a constant weight. The degradation rate was calculated using the following equation:$\text{ }\text{Weight loss }\left( \text{\%} \right)\text{=}\frac{\text{W}_{\text{0}}\text{ - }\text{W}_{\text{i}}}{\text{W}_{\text{0}}}\text{ × }\text{100\%}$

**Detailed animal experiment procedures**

Except for the negative control group, all rats received dural defect surgery. Before surgery, antibiotics were intramuscularly administered to prevent infection, and anesthesia was induced by intraperitoneal injection of 3% pentobarbital sodium (40 mg/kg). After scalp shaving, disinfection, and local anesthesia with 1% lidocaine, a midline frontal incision was made to expose the skull, followed by unilateral temporal craniotomy using a 1-cm cranial drill. Under a surgical microscope, an elliptical dura–arachnoid defect of approximately 0.8 × 0.8 cm was carefully created while preserving the cerebral cortex. The artificial dura substitute was trimmed to fit the defect and fixed to the dural margins with medical glue, followed by layered closure of the fascia and scalp with 3-0 sutures (Figure S1). Macroscopic and histopathological evaluations were focused on inflammation, brain injury, adhesion, connective tissue proliferation, fibrosis, vascular density, and material degradation, with statistical analysis conducted according to Table S1.

|  |  |  |  |
| --- | --- | --- | --- |
| **Skin incision** | **Removal of the craniotomy membrane** | **Drilling** | **Post-drilling** |
|  |  |  |  |
| **Removal of the cranial bone** | **Exposure of the dura mater** | **Excision of the dura mater** | **Implantation of the artificial dura mater** |
|  |  |  |  |
| **Implantation of the artificial dura mater** | **Fixation with medical adhesive** | **Suturing** | **Disinfection** |

**Figure S1:Modeling Procedure**

|  |  |  |  |
| --- | --- | --- | --- |
| **Negative control-7day** | **Posituve control-7day** | **CBPP-0-7day** | **CBPP-2-7day** |
|  |  |  |  |
| **Negative control-14day** | **Posituve control-14day** | **CBPP-0-14day** | **CBPP-2-14day** |
|  |  |  |  |
| **Negative control-28day** | **Positive control-28day** | **CBPP-0-28day** | **CBPP-2-28day** |

**Figure S2:Macroscopic Examination**

| Scoring types | Pathological features | **Score** | | | | |
| --- | --- | --- | --- | --- | --- | --- |
|  |  | **0** | **1** | **2** | **3** | **4** |
| Damage score | Degree of tissue adhesion/Brain damage | None | Narrow band | Moderate thickness band | Wide band | Broader band |
| Repair score | Dura mater formation | None | 1-25% bone resection area | 26%-50% bone resection area | 51%-75% bone resection area | More than 75% bone resection area |
|  | Microvessel density | None | Sparse and isolated microvessels | discrete capillary-like vessels are present within the regenerating tissue | Abundant microvessels distributed throughout most of the repair area | Dense and well-organized neovascular network |

**Table S1: Pathological Scoring Criteria**

**Figure S3. (A) TEM imaging and elemental analysis of bioactive glass in the electrospinning solution; (B) TEM morphology of CNPs; (C) XRD pattern of CNPs; (D) XPS spectra and valence state fitting of CNPs; (E) CCK-8 cytotoxicity assessment of CNPs.**

**Preparation and characterization of CNP**

CNP of different sizes and valence states were synthesized using a modified thermal decomposition method. Briefly, 10 mmol of Ce(NO3)3·6H2O (4.342 g), 150 mmol of 1-octadecene (39.455 g), and 30 mmol of oleylamine (8.025 g) were added to a three-neck round-bottom flask. The mixture was heated in an oil bath at 80 °C and stirred at 200 rpm for 30 min to completely dissolve the cerium precursor, forming a brownish-yellow solution. The temperature was then raised to 160 °C and maintained for 2 h to obtain the CNP-1. For the CNP-2 group, the same procedure was followed, with the addition of 400 mmol deionized water (7.2 g). The oleylamine concentration was subsequently reduced to 15 mmol (4.025 g) and 10 mmol (2.675 g) to produce CNP-3 and CNP-4, respectively. For CNP-5, nitrogen was continuously introduced during the synthesis of CNP-2 to provide an inert atmosphere. In the preparation of CNP-6, the reaction was carried out under nitrogen protection at 80 °C for 30 min, then heated to 190 °C and stirred for 2 h following the same procedure.

As shown in **Figure S3A**, the micrographs clearly revealed well-dispersed bioactive glass nanoparticles with diameters ranging from approximately 10 to 30 nm. In addition, salt-like precipitates were observed in close proximity to these nanoparticles. Elemental analysis indicated that the bioactive glass nanoparticles were primarily composed of silicon, phosphorus, and oxygen, with trace amounts of calcium and sodium. In contrast, the precipitated salts were mainly composed of calcium, sodium, silicon, and oxygen, with a relatively lower phosphorus content, suggesting that these deposits were calcium-sodium silicates and phosphates.

TEM analysis (**Figure S3B**) revealed that the particle sizes of the six CNP groups were 3.4 ± 0.3 nm, 3.4 ± 0.8 nm, 7.2 ± 0.1 nm, 10 ± 0.2 nm, 14 ± 1.6 nm, and 8.7 ± 0.7 nm, respectively, all exhibiting uniform size distribution. XRD patterns (Figure 1C) displayed characteristic diffraction peaks at 28.46° (111), 33.10° (200), 47.40° (220), 56.22° (311), 59.01° (222), and 69.69° (400), consistent with previously reported data, confirming the successful synthesis of cerium oxide nanoparticles (CNPs). Peak fitting of the XPS spectra (**Figure S3D**) was used to determine the oxidation states of cerium, revealing Ce³⁺ proportions of 33.73%, 56.65%, 35.00%, 53.21%, 22.42%, and 18.91% for CNP groups 1-6, respectively.

CNPs with varying particle sizes and valence states were ultrasonically dispersed in MEM medium containing 10% fetal bovine serum and 1% penicillin-streptomycin, maintaining a final nanoparticle concentration of 0.1%, and subsequently co-cultured with L929 cells. CCK-8 assay results showed that after 1, 3, and 5 days of incubation, all experimental groups exhibited cell viability above 90%, indicating no significant cytotoxicity at the 0.1% concentration. After one day of incubation, the CNP-5 group demonstrated the strongest promotion of cell proliferation, followed by the CNP-2 and CNP-6 groups. However, by days 3 and 5, the proliferation rates of the CNP-4, CNP-5, and CNP-6 groups dropped below that of the control, whereas CNP-2 maintained the highest proliferative effect. This suggests that after 24 h of co-culture, cells exposed to CNP-4, CNP-5, and CNP-6 experienced cytotoxic stress responses, leading to a sharp decline in proliferation over time. The reduced viability in the CNP-5 and CNP-6 groups may be attributed to their lower Ce³⁺ content, resulting in a weaker ability to regulate intracellular reactive oxygen species (ROS) levels. Although CNP-4 exhibited a higher Ce³⁺ ratio, its larger particle size (14 ± 1.6 nm) likely hindered cellular exchange with the surrounding medium, causing pronounced early cytotoxic stress and subsequent suppression of proliferation. Considering these factors, the CNP-2 group was selected for further experiments.

1. ^#^These authors contributed equally to the work.

   ^*^Corresponding author. E-mail address: lgy929@126.com (Guoyu Lv).
